# What is Known About Hospice Enrollment for Nursing Home Residents with Cognitive Disabilities: A Systematic Review

**DOI:** 10.1093/geroni/igaf122.2863

**Published:** 2025-12-31

**Authors:** Victoria Winogora, Patricia Stone, Kimberlee Grier, Lara Dhingra, Christine DeForge

**Affiliations:** Columbia University School of Nursing, New York, New York, United States; Columbia University, New York, New York, United States; Columbia University, New York, New York, United States; Metropolitan Jewish Health System, Inc., New York, New York, United States; Columbia University, New York, New York, United States

## Abstract

**Background:**

The population of individuals with cognitive disabilities (CD) accessing hospice care is increasing. CDs are defined as serious difficulty concentrating, remembering, and making decisions, and are often associated with neurocognitive disorders (e.g., dementias and Parkinson’s disease). Individuals with CDs often live and die in nursing homes (NHs). Little is known about hospice enrollment for individuals with CDs who die in NHs.

**Objective:**

To systematically review the evidence available on hospice enrollment in NHs for individuals with CDs.

**Methods:**

Following the PRISMA guidelines, we searched for United States-based, English-language, and peer-reviewed literature on hospice enrollment in NHs for individuals with CDs. PubMed, CINHAL, Web of Science, and Google Scholar were searched for articles published before September 20, 2024. The Joanna Briggs Institute Analytical Cross-Sectional Studies Appraisal Tool to assess study quality.

**Results:**

1,056 titles and abstracts were screened, 22 articles underwent full-text review, and 6 met inclusion criteria. The indicators of CD varied. In all studies, a significant percentage of NH decedents with CDs were not enrolled in hospice at the time of their deaths. Factors associated with increased likelihood of hospice enrollment at the time of death included female sex (n = 2), white race (n = 2), and non-CD terminal diagnosis (n = 1).

**Discussion:**

This systematic review is the first to explore hospice enrollment for NH residents with CDs. All studies focused on individuals with dementias. CD was associated with different rates of hospice enrollment for NH decedents. Future research should aim to explore interventions to improve hospice enrollment for this population.

